# Effects of glaucoma on detection and discrimination of image blur

**DOI:** 10.1111/opo.12947

**Published:** 2022-01-24

**Authors:** Habiba A Bham, Jonathan Denniss

**Affiliations:** ^1^ School of Optometry and Vision Science University of Bradford Bradford UK

**Keywords:** blur, detection, discrimination, glaucoma

## Abstract

**Purpose:**

Blur is one of the most commonly reported visual symptoms of glaucoma, but it is not directly measured by current clinical tests. We aimed to investigate the effects of glaucoma on detection and discrimination of image blur.

**Methods:**

Participants were people with glaucoma, separated into two groups with (*n* = 15) or without (*n* = 17) central visual field defects measured by 10–2 perimetry, and an age‐similar control group (*n* = 18). First, we measured contrast detection thresholds centrally using a 2‐interval forced choice procedure. We then measured blur detection and discrimination thresholds for the same stimuli (reference blurs 0, 1 arcmin) using a 2‐alternative forced choice procedure under two contrast conditions: 4× individual detection threshold for the low contrast condition; 95% contrast for the high contrast condition. The stimulus was a horizontal edge bisecting a hard‐edged circle of 4.5° diameter. Data were analysed by linear mixed modelling.

**Results:**

Contrast detection thresholds for the glaucoma group with central visual field defects were raised by 0.01 ± 0.004 (mean ± SE, Michelson units) (*p* = 0.002) and by 0.01 ± 0.004 (*p* = 0.03) relative to control and glaucoma without central visual field defect groups, respectively. Blur detection and discrimination thresholds were similar between groups, with small elevations in blur detection thresholds in the glaucoma groups not reaching statistical significance (detection *p* = 0.29, discrimination *p* = 0.91). The lower contrast level increased thresholds from the higher contrast level by 1.30 ± 0.10 arcmin (*p* < 0.001) and 1.05 ± 0.10 arcmin (*p* < 0.001) for blur detection and discrimination thresholds, respectively.

**Conclusions:**

Early‐moderate glaucoma resulted in only minimal elevations of blur detection thresholds that did not reach statistical significance in this study. Despite the prevalence of blur as a visual symptom of glaucoma, psychophysical measurements of blur detection or discrimination may not be good candidates for development as clinical tests for glaucoma.

**Key points**

- Early‐moderate glaucoma produces only minimal changes to blur detection and discrimination thresholds in central vision.
- Descriptions of ‘blur’ as a visual symptom in glaucoma may relate to non‐central vision, more advanced disease or be non‐specific to glaucoma.
- Psychophysical measurements of blur detection or discrimination are unlikely to be useful as clinical tests for glaucoma.

## INTRODUCTION

Glaucoma is characterised by loss of retinal ganglion cells which manifests clinically as localised reduction of contrast sensitivity, measured by perimetry. The visual experience of patients with glaucoma is not fully understood, and common depictions of glaucomatous vision loss often do not relate to the visual experiences of people with glaucoma.^1^ Recent studies have shown that one of the most common descriptions of visual symptoms provided by individuals with glaucoma is perception of blur.^1^, ^2^

In the healthy adult visual system, physiological blur is important for several visual functions, including as a cue for depth and motion.^3^, ^4^ Increases in blur can indicate a change in refractive error, the development of pathology impacting the optical quality of the eye such as cataract^5^, ^6^ or retinal pathology such as macular degeneration.^7^, ^8^, ^9^ Further, other normal visual phenomena including aliasing, crowding and spatial summation may be described as ‘blur’ by lay people. As such, the term ‘blur’ as used by lay people to describe their vision can be non‐specific, with many potential causes both pathological and physiological.

In glaucoma, it is evident that visual functions other than contrast sensitivity are impaired.^10^, ^11^, ^12^, ^13^, ^14^, ^15^, ^16^, ^17^, ^18^, ^19^, ^20^, ^21^ Notably, reduced spatial resolution,^11^ enlargement of the area of complete spatial summation^20^ and increased crowding^19^ have all been reported, and each are possible explanations for symptoms described as blur in the visual field. In non‐foveal vision, visual acuity may be limited by the density of retinal ganglion cells sampling the stimulus,^21^, ^22^ and as these cells are destroyed in glaucoma, the separation between functioning cells increases, reducing spatial resolution.^11^ Redmond et al.^20^ found an enlargement of the area of complete spatial summation in glaucoma that may be attributable to pooling of responses across more widespread retinal ganglion cells to maintain the overall neural signal,^23^ a process that may also contribute to increased crowding.^19^ Each of these factors is likely to reduce the ability to detect high spatial frequency image content, and therefore, produce the symptom of blur.

If the loss of retinal ganglion cells in glaucoma does cause an increase in neural blur, we would expect the ability to detect and discriminate image blur to be impaired as a result of the loss of ability to detect the high spatial frequency components that define the difference between images with different levels of blur. Therefore, the purpose of this study was to measure blur detection and discrimination in people with glaucoma and healthy control volunteers. We hypothesised that participants with glaucoma would have elevated thresholds for detection and discrimination of blur, particularly if the stimulus fell within a clinically‐measurable visual field defect. Since low contrast conditions increase blur thresholds in healthy vision,^24^ we further hypothesised that differences between glaucoma and healthy controls would be increased for lower contrast stimuli whereby there is a reduced probability of remaining retinal ganglion cells firing.

## METHODS

### Participants

Thirty‐two subjects with glaucoma (mean age 71, SD 6 years) and 18 age‐similar healthy control volunteers (mean age 70, SD 6 years) participated. Participants were recruited via local hospitals, community groups and advertisements in local newspapers. Diagnosis of glaucoma was confirmed by a clinical report from an ophthalmologist or a reliable self‐report with evidence of current treatment. For inclusion in the glaucoma group, we additionally required a visual field defect and at least one sectoral defect (p < 5%) of circumpapillary retinal nerve fibre layer thickness measured by optical coherence tomography (Spectralis; Heidelberg Engineering, heidelbergengineering.com). For this purpose, we defined a visual field defect as a cluster of three or more adjacent non‐edge points with pattern deviation p < 5% on a SITA Std 24–2 test (Humphrey Field Analyzer III; Carl Zeiss Meditec, zeiss.com).

**FIGURE 1:**
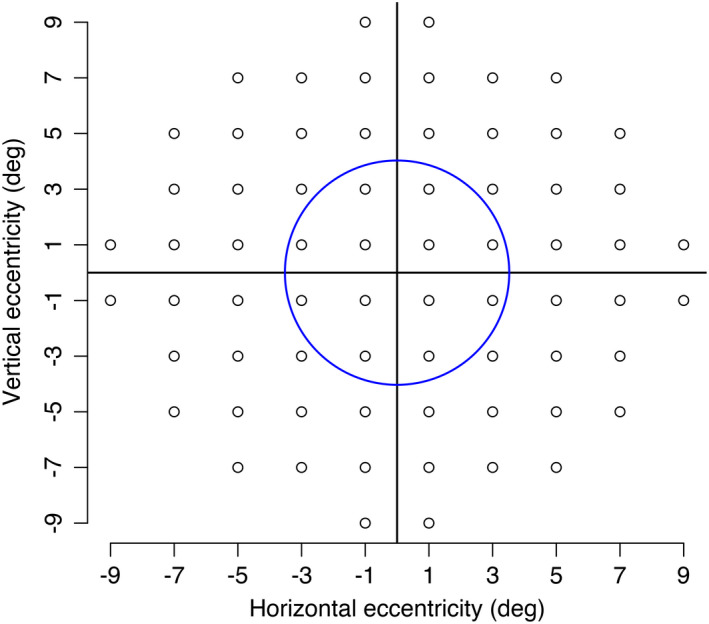
10–2 visual field locations. The circle encompasses the central 12 points used in the visual field criteria to define the two groups of glaucoma participants, with and without central visual field defects

Glaucoma participants were further divided into two groups: those with (glaucoma central visual field defect) and without (glaucoma normal central) central visual field defects. Central visual field defects were defined as three or more adjacent points with total deviation p < 5% within the central 12 points on a 10–2 SITA Standard test (Figure 1). This area of points was chosen as it covers a 6° diameter around fixation which most closely matches the size of the blur stimulus used (4.5°, see later).

Control participants were included in the study if they had no visual field defect on either 24–2 or 10–2 SITA Standard tests, with Glaucoma Hemifield Test results ‘within normal limits’. No optical coherence tomography inclusion/exclusion criterion was applied to the control group.

All participants had a monocular visual acuity of better than 0.2 logMAR (6/9.5) in the tested eye and a refractive error not more than 6.00DS and 3.00DC. Visual acuity was measured using an electronic logMAR chart at 6 m while wearing appropriate refractive correction for this viewing distance. Participants were only included in the study if they had no ocular or systemic condition known to affect visual performance except glaucoma for the glaucoma group, and mild cataract (no more than NC3 NO3 C2 P2 on the LOCS III grading scale^25^). Control subjects were required to have normal ocular health findings including slit‐lamp biomicroscopy, indirect fundoscopy and Goldmann applanation tonometry (intraocular pressure ≤21 mmHg and ≤3 mmHg difference between the eyes).

Testing was conducted monocularly using the best refractive correction for the screen distance as determined by subjective refraction by an optometrist. If both eyes fitted the inclusion criteria for any participant, the tested eye was chosen at random. All participants provided written informed consent in accordance with the tenets of the Declaration of Helsinki before participating in the study. The study received ethics approval from the National Health Service (NHS) Research Ethics Service. An inconvenience allowance was provided to participants.

### Apparatus and stimuli

**FIGURE 2:**
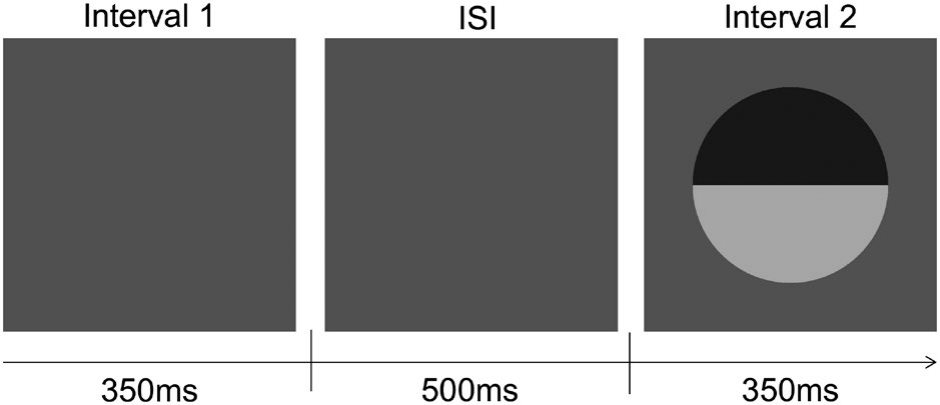
Two‐interval forced‐choice procedure used to measure contrast detection thresholds. In each trial, stimuli were presented randomly in either interval 1 or 2. Interval duration was 350 ms with an inter‐stimulus interval (ISI) duration of 500 ms. Participants indicated which interval the stimulus appeared in by pressing a key

Blur detection and discrimination were measured using a horizontal edge bisecting a hard‐edged circle of 4.5° diameter (see Figure 2, rightmost panel). Contrast was defined in Michelson units as (L_max_ − L_min_)/(L_max_ + L_min_), where L_max_ and L_min_ are the maximum and minimum luminance of the stimulus, respectively. Stimulus contrast was set individually for each participant for the low contrast condition (see Procedure section) and at 95% contrast for the high contrast condition. The horizontal edge was blurred by a Gaussian kernel of varying spread that operated as a low‐pass spatial filter. Stimulus blur was defined by the spread (standard deviation) of this blurring kernel, reported in arcmin. Two reference blurs were used; 0 arcmin for blur detection and 1 arcmin for blur discrimination. A third reference blur of 4 arcmin was incorporated into the contrast detection task but not used for subsequent blur detection and discrimination tasks, as pilot testing showed the task to be too difficult for participants at this reference blur. Stimuli were generated in MATLAB version 8.5.0 (R2015a; MathWorks, mathworks.com) using Psychtoolbox‐3 version V3.0.14).^26^, ^27^, ^28^ Stimuli were presented on a 14 bit calibrated display system (resolution 1920 × 1080, refresh rate 120 Hz; CRS Display++; Cambridge Research Systems, crsltd.com) viewed from 127 cm while using a chin and forehead rest. Appropriate refractive correction was worn for this viewing distance and testing was performed monocularly with occlusion of the non‐tested eye. The mean luminance of the screen was 52.8 cd/m².

### Procedure

#### Measurement of contrast detection thresholds

We first measured detection thresholds in order to determine appropriate contrasts for the low contrast condition of the blur detection and discrimination measurements, which was individually set for each participant. Contrast detection thresholds were obtained through a two‐stage process. First, approximate thresholds were determined using the method of adjustment. Participants fixated on the stimulus (reference blur 0, 1 or 4 arcmin) presented centrally, and were instructed to adjust the contrast of the stimulus using a dial (CB7, Cambridge Research Systems, crsltd.com) until they could ‘just see it’. A rotation of the dial clockwise or anti‐clockwise resulted in an increase or decrease in contrast, respectively. One full rotation of the dial produced a 10% change in contrast. The resulting threshold approximations were then used as a starting point for the second task to obtain final detection thresholds.

Final contrast detection thresholds were then obtained using a 2‐interval forced choice (which interval?) procedure. Stimuli were presented for 350 ms with a raised cosine temporal profile, separated by a 500 ms inter‐stimulus interval (Figure 2). Stimulus contrast was adjusted according to a three‐down, one‐up (3D1 U) staircase procedure with independent staircases randomly interleaved for each reference blur (0, 1 and 4 arcmin). Trials with stimuli of 95% contrast were included for the first two presentations and then every tenth presentation to help maintain attention. Step sizes for stimulus contrast adjustment were 20% before the first reversal and 10% thereafter. Staircases terminated after six reversals, with the mean of the last four being taken as the detection threshold. Participants were instructed to identify whether the stimulus appeared in interval 1 or 2 and to give their best guess when they were unsure. Responses were recorded by pressing a key.

#### Measurement of blur detection and discrimination thresholds

**FIGURE 3:**
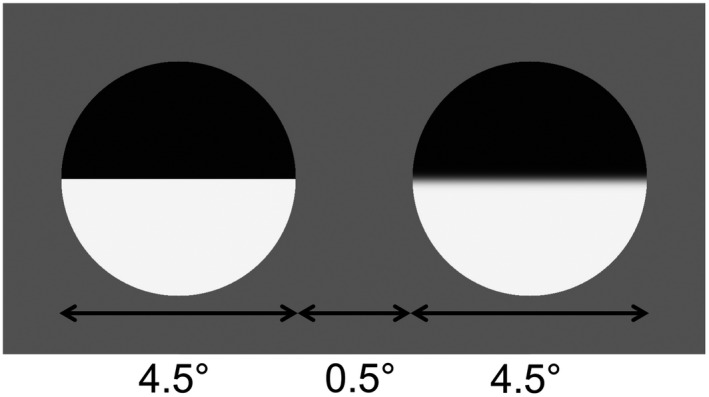
Stimulus arrangement for the free viewing, 2‐alternative forced‐choice procedure used to measure thresholds for blur detection (reference blur 0 arcmin) and discrimination (reference blur 1 arcmin). Reference and test stimuli were presented randomly either to the left or right of the centre of screen. Participants were instructed to indicate which of the two stimuli was sharper

Blur detection and discrimination thresholds were measured centrally for stimuli of reference blur 0 and 1 arcmin using a 2‐alternative forced choice (which is sharper?) procedure. Stimuli were presented side by side with their edges separated by 0.5° under free viewing conditions (Figure 3). The reference stimulus had the reference blur (r) whilst the test stimulus had blur equal to the reference +a blur increment (r + Δr). The reference and test stimuli were presented randomly between the two positions on screen, centred either 2.5° left or 2.5° right from the centre of the screen. Participants were instructed to identify which of the two stimuli (left/right) was clearer and to give their best guess if unsure. Responses were recorded by pressing a key. Stimuli were presented for 1200 ms with a raised cosine temporal profile and an inter‐trial interval of 500 ms. Trials displaying a test blur of 10 arcmin were included for the first two presentations and then every tenth presentation thereafter to help maintain attention.

A total of four test conditions were examined: blur detection (reference blur 0 arcmin) and discrimination (reference blur 1 arcmin), each under two contrast conditions, 4× individual contrast detection threshold for the low contrast condition and 95% contrast for the high contrast condition. Participants performed these tasks in a predetermined randomised order. Test blur increment was varied according to three independent randomly interleaved 3D1 U staircases. Each staircase began randomly from a blur increment of 2–6 arcmin. This start blur increment was chosen based on previous pilot data collected from young and older healthy participants. Test blur increment was increased or decreased in 20% step sizes before the first reversal and 10% thereafter. Staircases terminated after eight reversals and thresholds were calculated as the average of all but the first two reversals.

If staircases did not converge or data were of poor quality, the experimental run was repeated and blur increments between 0–10 arcmin for the start of staircases were manually selected. These start levels were chosen to begin at or above the level of staircase termination from the previous run depending on data quality and convergence of staircases. If data quality was good and staircases showed convergence but did not quite converge, staircases began at a similar level from where the previous staircases terminated. However, if data quality was poor and staircases were not converging, the manual start level of staircases began higher than where previous staircases terminated, as blur increments appeared not to be sufficiently above threshold for the participant to perform the task appropriately. A maximum of three runs per condition were completed.

### Statistical analysis

Statistical analysis was completed in *R* ^29^ using the lme4^30^ and emmeans^31^ packages. To determine between‐group and between‐stimulus differences we used linear mixed modelling, comparing models with chi squared likelihood ratio tests. For contrast detection data, fixed effects of group (glaucoma central VF defect, glaucoma normal central, controls) and reference blur (0, 1, 4 arcmin) and random effects of participant were entered into the model. Models took the following forms, computed sequentially:

$$\text{Null}:\;\text{Contrast}\;\text{detection}\;\text{threshold}∼1+\left(1|\text{Participant}\right)$$

$$\begin{array}{c} \text{Fixed}‐\text{effect}\;\text{of}\;\text{Group}:\text{Contrast}\;\text{detection}\;\text{threshold}∼1\\ +\text{Group}+\left(1|\text{Participant}\right) \end{array}$$

$$\begin{array}{c} \text{Fixed}‐\text{effect}\;\text{of}\;\text{Reference}\;\text{blur}:\text{Contrast}\;\text{detection}\;\text{threshold}∼1\\ +\text{Group}+\text{Reference}\;\text{Blur}+\left(1|\text{Participant}\right) \end{array}$$

In the models, 1 denotes the intercept, with 1|Participant indicating random intercepts for each participant. In the final model, the fixed effect of group was only included if a likelihood ratio test showed a statistically significant effect (*p* < 0.05) in the previous comparison (fixed effect of group vs. null).

For blur detection and discrimination data, fixed effects of group (glaucoma central VF defect, glaucoma normal central, controls) and contrast level (4× detection threshold, 95% contrast) and random effects of participant were entered into the model. Models took the following forms, computed sequentially:

$$\text{Null}:\text{Blur}\;\text{threshold}∼1+\left(1|\text{Participant}\right)$$

$$\begin{array}{c} \text{Fixed}‐\text{effect}\;\text{of}\;\text{Group}:\text{Blur}\;\text{threshold}∼1\\ +\text{Group}+(1|\text{Participant}) \end{array}$$

$$\begin{array}{c} \text{Fixed}‐\text{effect}\;\text{of}\;\text{Contrast}:\text{Blur}\;\text{threshold}∼1\\ +\text{Contrast}+(1|\text{Participant}) \end{array}$$

Null models were compared to alternative models including the fixed‐effect in question using χ^2^ likelihood ratio test. If likelihood ratio tests were significant (*p* < 0.05), Tukey post‐hoc test using estimated marginal means separated effects by group/reference blur/contrast and calculated effect sizes was employed. Interaction effects were also assessed between group and reference blur (contrast detection data) and group and contrast (blur detection and discrimination data) using the same approach.

## RESULTS

From the total 32 glaucoma participants, 15 were assigned to the glaucoma central VF defect group (Table 1), whilst 17 were assigned to the glaucoma normal central group (Table 2).

**TABLE 1:** Individual visual field summary indices, visual acuity and cataract grading for the tested eye of participants in the glaucoma central visual field (VF) defect group

Participant	Age (years)	24–2 MD (dB)	24–2 PSD (dB)	24–2 GHT	10–2 MD (dB)	10–2 PSD (dB)	BCVA (logMAR)	LOCSIII grading
1	75	−2.57	6.27	ONL	−4.71	6.59	0.12	Clear IOL
2	64	−5.64	7.63	ONL	−7.23	8.77	0.10	NC0.5 NO0.5 C0 P0
3	65	−2.69	10.70	ONL	−12.51	16.37	0.00	NC1 NO1 C0 P0
4	77	−3.33	6.32	ONL	−6.25	10.35	0.00	NC2 NO2 C0 P0
5	61	−14.95	10.43	ONL	−11.44	10.60	−0.02	NC2 NO2 C0 P0
6	67	−8.62	10.11	ONL	−14.92	14.97	0.10	NC1.5 NO1.5 C0 P0
7	66	−1.92	5.04	ONL	−7.54	11.09	0.08	NC2 NO2 C0 P0
8	77	−12.77	13.09	ONL	−11.50	13.89	0.12	Clear IOL
9	84	−4.34	5.92	ONL	−5.79	8.82	0.12	Clear IOL
10	78	−4.53	2.48	GRS	−3.82	1.71	0.04	Clear IOL
11	74	−13.08	10.68	ONL	−5.43	3.18	0.02	NC2 NO2 C0 P0
12	76	−8.00	4.25	ONL	−8.65	7.70	0.02	Clear IOL
13	64	−4.50	10.44	ONL	−14.00	16.83	0.02	NC1.5 NO1.5 C0 P0
14	70	−3.21	2.48	ONL	−5.10	1.97	−0.10	Clear IOL
15	69	−4.83	2.72	BL/GRS	−4.04	3.73	0.10	NC1 NO1 C0 P0

Abbreviations: BCVA, Best Corrected Visual Acuity; BL, Borderline; GHT, Glaucoma Hemifield Test; GRS, Generalised Reduction of Sensitivity; IOL, intraocular lens; LOCSIII, Lens Opacity Classification System III; MD, Mean Deviation; ONL, Outside Normal Limits; PSD, Pattern Standard Deviation.

**TABLE 2:** Individual visual field summary indices, visual acuity and cataract grading for the tested eye of participants in the glaucoma normal central group

Participant	Age (y)	24–2 MD (dB)	24–2 PSD (dB)	24–2 GHT	10–2 MD (dB)	10–2 PSD (dB)	BCVA (logMAR)	LOCSIII grading
1	73	−4.71	6.15	ONL	−3.55	1.68	−0.04	Clear IOL
2	78	−6.13	6.04	ONL	−3.03	1.60	0.10	Clear IOL
3	70	−2.35	3.66	ONL	−0.47	1.49	−0.08	NC1.5 NO1.5 C0 P0
4	61	−6.29	11.17	ONL	−9.35	15.03	0.04	NC1 NO1 C0 P0
5	70	−3.01	2.20	ONL	−2.95	4.04	−0.02	NC1 NO1 C0 P0
6	74	−1.46	2.77	ONL	−4.72	9.26	0.06	Clear IOL
7	67	−5.91	6.57	ONL	−2.49	2.33	0.06	NC1 NO1 C0 P0
8	69	−1.61	6.50	ONL	0.79	1.74	−0.06	NC2.5 NO2.5 C0 P0
9	69	−3.20	3.05	ONL	−1.97	2.22	0.06	NC1.5 NO1.5 C0 P0
10	73	−5.69	7.18	ONL	−2.78	1.75	0.10	NC2.5 NO2.5 C0.5 P0.5
11	83	−3.71	2.34	WNL	−1.57	1.34	0.12	Clear IOL
12	74	−5.60	10.60	ONL	−10.02	14.32	0.10	Clear IOL
13	62	−4.06	8.38	ONL	−9.59	11.47	0.10	NC1 NO1 C0 P0
14	61	−5.72	9.61	ONL	−2.82	7.48	−0.10	NC1 NO1 C0 P0
15	69	−2.70	2.23	BL	−3.73	1.92	0.08	NC2 NO2 C0 P0
16	76	−4.38	7.18	ONL	−1.07	1.86	0.04	NC2.5 NO2.5 C0 P0
17	73	−2.95	4.90	ONL	−1.77	1.84	0.02	NC1 NO1 C0 P0

Note

Abbreviations as in Table 1.

Abbreviation: WNL, within normal limits.

Datasets where staircases did not converge even after multiple runs were excluded from analyses. This applied to a total of seven datasets, a breakdown of which is given in Table 3. Two glaucoma participants with central visual field defects could not complete these blur experiment tasks accurately enough for all four conditions, and so these two participants' datasets were completely removed and not included within the blur analysis. These two participants are not included in the tables or the numbers above.

**TABLE 3:** Number of datasets removed for each experimental condition due to non‐convergent staircases

	Ref Blur 0, Low contrast	Ref Blur 0, High contrast	Ref Blur 1, Low contrast	Ref Blur 1, High contrast
Controls	3	0	0	1
Glaucoma normal central VF	0	0	1	0
Glaucoma central VF defect	0	1	1	0

Abbreviation: VF, visual field.

### Contrast detection thresholds

**FIGURE 4:**
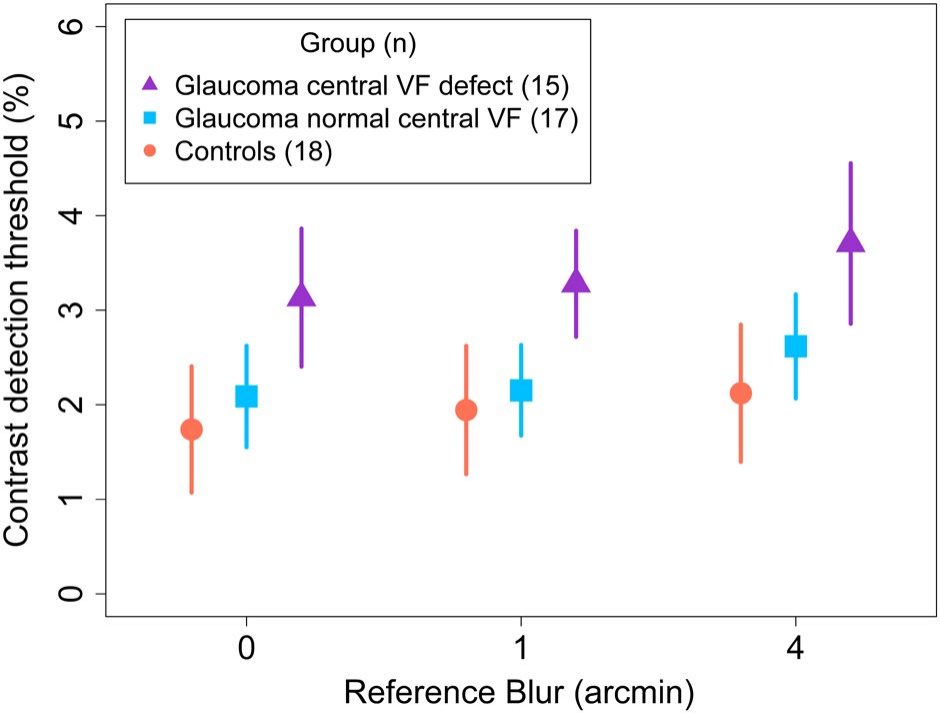
Contrast detection thresholds for controls (orange circles), glaucoma participants with a central visual field defect (purple triangles), and glaucoma participants without a central visual field defect (blue squares). Thresholds are given as Michelson units converted into percentages (Michelson units × 100). Data shown are mean ± 95% CI (confidence interval)

Group mean contrast detection thresholds for each reference blur are shown in Figure 4. Contrast detection thresholds differed between groups (main effect, χ^2^(2) = 12.6, *p* = 0.002). Specifically, detection thresholds for the glaucoma central VF defect group were elevated by 0.01 ± 0.004 (*p* = 0.002) (mean ± SE) and by 0.01 ± 0.004 (*p* = 0.03) relative to controls and glaucoma normal central groups, respectively. Contrast detection thresholds were similar between control and glaucoma normal central groups (difference in mean detection thresholds, 0.004 ± 0.004, *p* = 0.6).

Contrast detection thresholds were significantly affected by reference blur (χ^2^(2) = 20.85, *p* < 0.001). Specifically, contrast detection thresholds were raised for a reference blur of 4 arcmin by 0.005 ± 0.001 (*p* < 0.001) and by 0.004 ± 0.001 (*p* = 0.003) relative to reference blurs of 0 and 1 arcmin, respectively. Contrast detection thresholds were similar between reference blurs 0 and 1 arcmin (difference in mean detection thresholds, 0.001 ± 0.001, *p* = 0.40). No significant interaction was found between group and reference blur (χ^2^(4) = 1.87, *p* = 0.76).

### Blur detection thresholds

**FIGURE 5:**
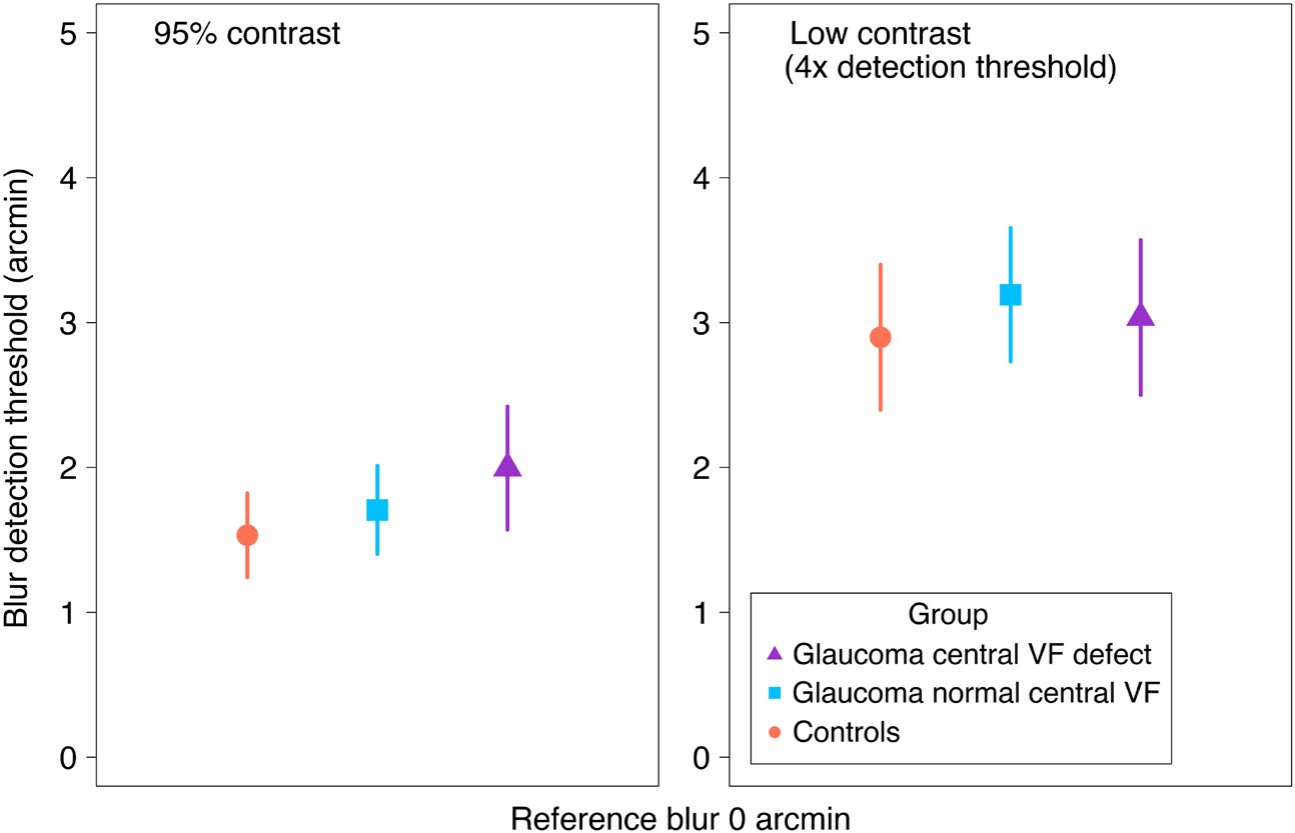
Blur detection thresholds (mean ± 95% confidence interval [CI] of the mean) for each group for high (left) and low (right) contrast stimuli. Symbols as in Figure 4

Figure 5 shows group mean blur detection thresholds for high (95%) and low (4× detection threshold) contrast levels. Overall, blur detection thresholds were similar across the three groups (main effect of group, χ^2^(2) = 2.46, *p* = 0.29) with group means ± SE of 2.15 ± 0.18, 2.45 ± 0.18 and 2.53 ± 0.19 arcmin for control, glaucoma normal central and glaucoma central VF defect groups, respectively. There was a main effect of contrast level on blur detection thresholds (χ^2^(1) = 73.49, *p* < 0.001). Specifically, the lower contrast level (4× detection threshold) increased blur detection thresholds by 1.3 ± 0.10 arcmin compared with the higher (95%) contrast level.

A slightly different, though not quite statistically significant, trend in blur detection threshold elevation with change in contrast (Figure 5) was observed between the three groups (interaction between group and contrast level, χ^2^(2) = 5.94, *p* = 0.05). Specifically, a decrease in contrast from 95% contrast to 4× detection threshold increased blur detection thresholds by 1.42 ± 0.17, 1.49 ± 0.16 and 0.94 ± 0.17 arcmin for control, glaucoma normal central and glaucoma central VF defect groups, respectively.

### Blur discrimination thresholds

**FIGURE 6:**
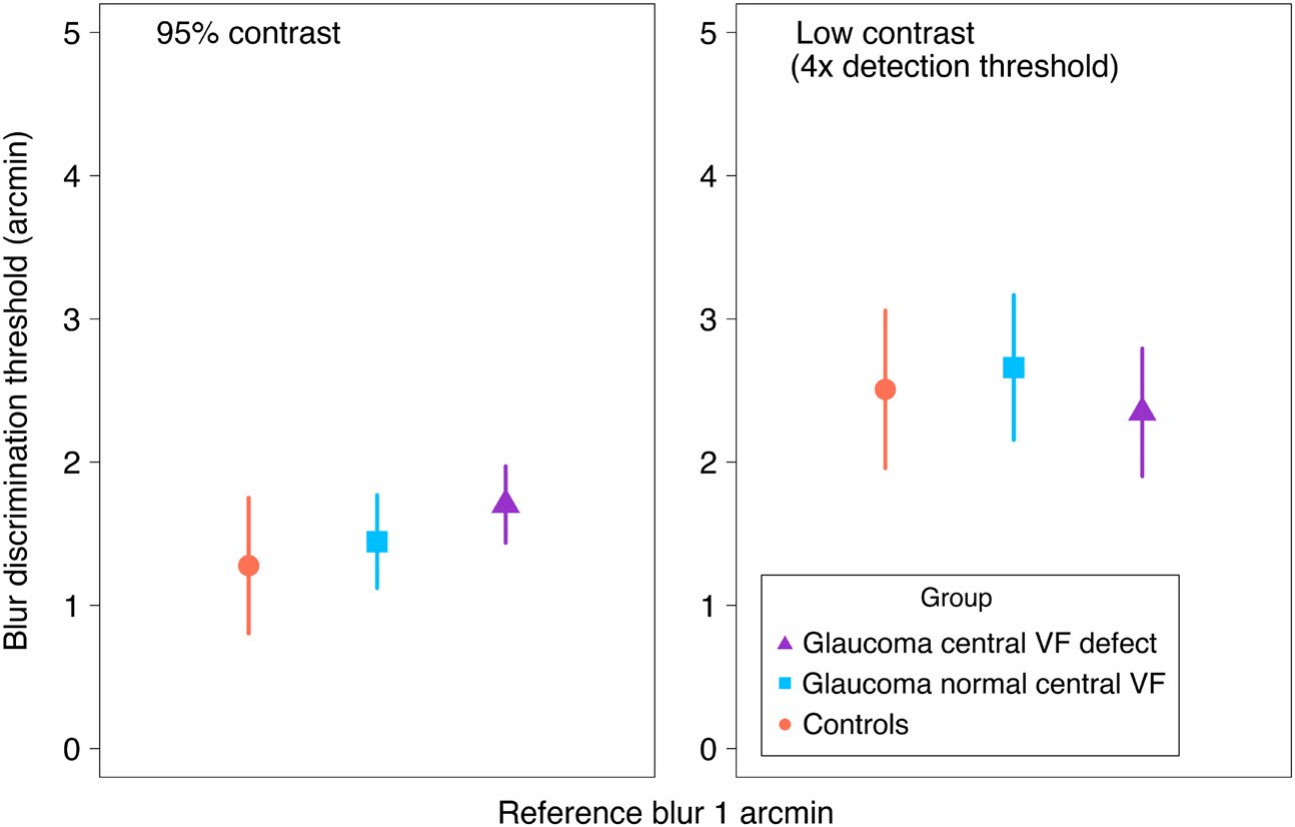
Group blur discrimination thresholds (mean ± 95% confidence interval [CI] of the mean) for a reference blur of 1 arcmin for high (left) and low (right) contrast stimuli. Symbols as in Figure 4

Figure 6 shows group mean blur discrimination thresholds for a reference blur of 1 arcmin for both high (95%) and low (4× detection threshold) contrast stimuli. Blur discrimination thresholds were similar across groups (main effect of group, χ^2^(2) = 0.18, *p* = 0.91) with group means ± SE of 1.92 ± 0.19, 2.02 ± 0.19 and 2.02 ± 0.21 arcmin for control, glaucoma normal central and glaucoma central VF defect groups, respectively. There was a main effect of contrast level on blur discrimination thresholds (χ^2^(1) = 60.26, *p* < 0.001). Similar to the detection condition, the lower contrast level (4× detection threshold) increased blur discrimination thresholds by 1.05 ± 0.096 arcmin compared with 95% contrast.

For blur discrimination, there was an interaction between group and contrast level (χ^2^(2) = 7.55, *p* = 0.02). A decrease in stimulus contrast from 95% contrast to 4× detection threshold increased blur discrimination thresholds to a greater extent for the glaucoma normal central and control groups compared with the glaucoma VF defect group (Figure 6). Specifically, this decrease in contrast level increased mean blur discrimination thresholds by 1.17 ± 0.15 arcmin for the control group and by 1.26 ± 0.16 arcmin for the glaucoma normal central group, compared with 0.68 ± 0.17 arcmin for the glaucoma central VF defect group (*p* = 0.08 and *p* = 0.04, respectively, compared to glaucoma central VF defect group). Changes in blur discrimination thresholds with respect to changes in contrast were similar between glaucoma normal central and control groups (*p* = 0.91).

## DISCUSSION

The purpose of this study was to test the hypotheses that glaucoma increases blur detection and discrimination thresholds relative to controls, and that the effect would be greater under low contrast conditions. Our data showed only small increases in blur detection and discrimination thresholds in participants with glaucoma compared to age‐similar healthy individuals that did not reach statistical significance in this sample. Reducing stimulus contrast did increase blur detection and discrimination thresholds for all groups, in line with previous studies of healthy individuals.^24^, ^32^ Contrary to our hypothesis, reducing stimulus contrast elevated blur discrimination thresholds to a greater extent for glaucoma normal central and control groups compared with the glaucoma central VF defect group. Together, these results suggest that in early to moderate glaucoma, impairments in detection and discrimination of image blur in central vision are mild, even in the presence of clinically measurable visual field defects in the area of the stimulus. These results are consistent with the often symptomless nature of early glaucoma.

The smaller than expected increase in blur detection and discrimination thresholds in glaucoma with a central visual field defect may be explained by the optical limitations on resolution in central vision. Due to the dense neural sampling in the central retina, resolution in this area is thought not to be sampling limited, but limited by the eye's optics.^21^ Glaucoma may well reduce retinal ganglion cell sampling density in central vision, increasing neural blur, but this may not manifest as an impairment to blur detection or discrimination ability until the neural blur exceeds the optical blur. As such, the effects of early glaucomatous damage in the central retina on the present tasks may be partially masked by optical blur, reducing the apparent effect of glaucoma.

The observed interactions between contrast and group on blur discrimination thresholds were contrary to our initial hypothesis that there would be greater elevations of blur detection and discrimination thresholds under reduced contrast for the glaucoma group. In fact, our data showed less threshold elevation for the glaucoma central VF defect group, particularly for the discrimination condition. At high contrast, the glaucoma central VF defect group performed slightly worse at blur detection and discrimination compared to the other two groups, although this difference was small and not statistically significant for blur detection. However, at low contrast all three groups had similar blur detection and discrimination thresholds resulting in the glaucoma central VF defect group being less affected by a change in contrast than the other two groups. Low contrast stimuli contain less energy at all spatial frequencies then equivalent high contrast stimuli, but the reduction in energy at the high spatial frequencies that define stimulus sharpness may render these image components sub‐threshold for all observers due to the high spatial frequency drop‐off of the human contrast sensitivity function. This effect may partially mask losses of high spatial frequency sensitivity due to neural loss in glaucoma, such that already slightly elevated thresholds (Figures 5 and 6) are not further elevated as much as for healthy controls.

An alternative potential explanation for the approximately equal blur detection and discrimination thresholds for low contrast stimuli (Figures 5 and 6) may be related to our choice to set the reduced contrast to 4× the individual's contrast detection threshold. Since the glaucoma central VF defect group had higher contrast detection thresholds, the low contrast stimuli had higher physical contrast for this group than for the glaucoma normal central group, whose stimuli, in turn, had higher physical contrast than the control group's stimuli. Our recent study of suprathreshold contrast perception in early‐moderate glaucoma^12^ showed that individuals with glaucoma perceive the contrast of suprathreshold stimuli similarly to those with healthy vision, despite elevation of threshold contrast sensitivity. One possible explanation for that finding is a mechanism that alters contrast gain to compensate for neural losses, maintaining the overall signal. If such a mechanism exists, blur detection/discrimination thresholds may be linked to physical stimulus contrast, rather than how far above threshold the contrast is. This could explain our results as the loss of detection/discrimination ability may be compensated for by increases in physical contrast of the stimulus in our experiment.

Based on the hypotheses above, it follows that a more manifest elevation in blur detection/discrimination thresholds due to glaucoma may be present in more peripheral vision, where resolution is thought to be limited by the sampling density of retinal ganglion cells.^33^ However, pilot testing using the same stimulus presented at 5° or 9° eccentricity in a two interval forced‐choice paradigm showed that the task was too difficult for the participants to perform in non‐central vision, yielding mostly unreliable data. Given the small differences found between the glaucoma central VF defect and control groups in central vision, and the difficulty of obtaining measurements in non‐central vision where larger differences may be manifest, it seems unlikely that blur‐based psychophysical tests will prove useful for clinical detection or monitoring of glaucoma.

Comparing blur thresholds between different studies can be difficult due to differences in stimuli, participants and test procedure. Nevertheless, when compared with previous studies investigating blur detection and discrimination in healthy and usually younger individuals (see Watson and Ahumada^34^ for a comprehensive review), we see an elevation in blur thresholds in our data. For instance, blur detection and discrimination thresholds for 80% contrast stimuli have been found as 0.4 to 0.9 arcmin for blur detection and 0.15 to 0.4 arcmin for blur discrimination with reference blur 1 arcmin.^3^, ^35^ The nearest equivalent thresholds in our older healthy control participants were 1.53 ± 0.29 arcmin and 1.28 ± 0.47 arcmin. Similarly, for stimuli of lower contrast, our thresholds for healthy participants were 2.90 ± 0.5 arcmin and 2.51 ± 0.55 arcmin compared with previous studies showing these thresholds as 1–1.4 arcmin and 0.4–0.9 arcmin for stimuli of 10% contrast, which is broadly comparable to the contrast in our 4× contrast detection threshold condition.^3^, ^36^ These differences may be wholly or partly attributed to differing psychophysical procedures and stimuli. For example Wuerger et al.,^36^ and Mather and Smith^3^ used a 2‐interval forced choice task as opposed to the 2‐alternative forced choice free‐viewing paradigm used in this study. Mather and Smith^3^ used a larger square stimulus with a vertical and horizontal length of 8.72° and a sinusoidal edge whilst the present study used a smaller sized circular stimulus of 4.5° diameter and a straight edge. However, the difference in results may not be fully accounted for by stimulus and procedural differences alone, and may evidence an age‐related decline in these blur detection and discrimination thresholds. These findings may be attributed to the deterioration of optical factors in the eye that are found with ageing,^37^ but are not impacted by glaucoma. Although the point spread function for Gaussian blur is different to that derived from dioptric blur,^38^ it is of clinical relevance to consider the dioptric equivalence of the present blur thresholds to give a more clinically‐relatable description. Using the blur disc diameter equation: b° = 0.057pD, where b is the blur disc diameter in degrees of visual angle, p is pupil size in millimetres and D is dioptric blur, our measured blur detection/discrimination thresholds are approximately equivalent to 0.25D for low contrast stimuli and 0.15D for high contrast stimuli (pupil size‐2–4 mm).

The ability of the present study to identify subtle between‐group differences with statistical significance is limited by the sample size. Figures 5 and 6 both show such subtle differences, but these were not statistically significant at *p* < 0.05. While a larger sample size would likely render these subtle differences statistically significant, our current estimates of effect size indicate that between‐group differences are unlikely to be large and clinically useful for diagnosis or monitoring of glaucoma. The use of a free viewing paradigm may also be seen as a limitation since it could enable the participants to view the edge stimulus using regions of vision away from their visual field defect. Nevertheless, this approach still requires the participant to either use more peripheral vision, where performance would be impaired, or to attend to only a portion of the edge. We are not aware of data on blur detection/discrimination performance when only attending to a portion of the stimulus, but would hypothesise that this too would impair performance. The stimulus size for this study was chosen to be large enough to make the task manageable for the participants, but small enough to be localised to within visual field defects measured on the 10–2 visual field test.

The results of this study suggest that impairments to blur detection and discrimination in central vision due to glaucoma are small, and are most apparent under high contrast conditions. Patient reports of blur as a visual symptom of glaucoma may relate to more peripheral vision, or to more advanced stages of damage. Given the difficulty of performing the task and the small effect of early glaucoma, psychophysical tests of blur detection or discrimination similar to those employed in this study are unlikely to be useful as clinical diagnostic tests, despite the prevalence of blur as a visual symptom of glaucoma.

## CONFLICT OF INTEREST

The authors report no conflicts of interest and have no proprietary interest in any of the materials mentioned in this article.

## AUTHOR CONTRIBUTIONS

**Habiba A Bham:** Conceptualization (supporting); Data curation (lead); Formal analysis (lead); Investigation (lead); Methodology (supporting); Project administration (lead); Software (supporting); Validation (lead); Visualization (lead); Writing – original draft (lead); Writing – review & editing (supporting). **Jonathan Denniss:** Conceptualization (lead); Formal analysis (supporting); Funding acquisition (lead); Investigation (supporting); Methodology (lead); Project administration (supporting); Resources (lead); Software (lead); Supervision (lead); Validation (supporting); Visualization (supporting); Writing – review & editing (lead).
